# Lightweight Driver Behavior Identification Model with Sparse Learning on In-Vehicle CAN-BUS Sensor Data

**DOI:** 10.3390/s20185030

**Published:** 2020-09-04

**Authors:** Shan Ullah, Deok-Hwan Kim

**Affiliations:** Department of Electronic Engineering, Inha University, Incheon 22212, Korea; shan.ullah@iesl.inha.ac.kr

**Keywords:** driver-behavior identification, deep learning, Jetson Xavier, network pruning, sparse learning, convolutional neural network (CNN), long short-term memory (LSTM), edge computing

## Abstract

This study focuses on driver-behavior identification and its application to finding embedded solutions in a connected car environment. We present a lightweight, end-to-end deep-learning framework for performing driver-behavior identification using in-vehicle controller area network (CAN-BUS) sensor data. The proposed method outperforms the state-of-the-art driver-behavior profiling models. Particularly, it exhibits significantly reduced computations (i.e., reduced numbers both of floating-point operations and parameters), more efficient memory usage (compact model size), and less inference time. The proposed architecture features depth-wise convolution, along with augmented recurrent neural networks (long short-term memory or gated recurrent unit), for time-series classification. The minimum time-step length (window size) required in the proposed method is significantly lower than that required by recent algorithms. We compared our results with compressed versions of existing models by applying efficient channel pruning on several layers of current models. Furthermore, our network can adapt to new classes using sparse-learning techniques, that is, by freezing relatively strong nodes at the fully connected layer for the existing classes and improving the weaker nodes by retraining them using data regarding the new classes. We successfully deploy the proposed method in a container environment using NVIDIA Docker in an embedded system (Xavier, TX2, and Nano) and comprehensively evaluate it with regard to numerous performance metrics.

## 1. Introduction

Over the years, machine-learning algorithms have revolutionized the human lifestyle. Additionally, the continued evolution of hardware technologies (e.g., multi-core CPUs and GPUs in compact devices) has increased the benefits of intelligent algorithms in terms of their deployment in industries. During the past decades, the automobile industry has accelerated toward the future vehicle technology, making cars smarter and more connected than before. In addition to advanced mechanical components, modern vehicles are replete with multiple embedded computers that are responsible for vehicle control, safety features, and infotainment. The connectivity of cars via Wifi and 3G/4G wireless communication, as well as the intelligent embedded systems inside cars, has opened opportunities for employing cloud-based services and edge computing in automobiles. In a connected car environment, the driver can access his/her car, which is further connected to the edge server that provides all driving-related services. These services include insurance services [1,2] by awarding scores to the drivers, providing traffic information, providing optimal route information [3] (based on fuel efficiency and shortest distance), manufacturer services [4] (e.g., predictive vehicle maintenance), and monitoring services. The increasing number of in-vehicle sensors and their connectivity with the driver and other vehicles via edge and cloud for multiple services is increasing the comfort level of society. However, the continuous connectivity of connected vehicles entails security concerns, rendering cars more vulnerable to hacking and theft [5,6]. This has attracted the continuous attention of researchers toward driver-behavior profiling for identifying [5,7,8] the driver for several other applications.

The behavior of a driver can be characterized by analyzing the unique features associated with his/her driving skills and habits. The driving information is usually acquired using in-vehicle sensors [9,10] or sometimes smartphone-based sensors [9,11]. The in-vehicle controller area network-BUS (CAN-BUS) data include the information corresponding to the steering wheel, vehicle speed, brake-pedal position, and so forth, and the smartphone sensor data include the information related to speed, orientation, and acceleration. Recently, the in-vehicle CAN-BUS data have been regarded as accurate and reliable for driver profiling [10]; moreover, they have been analyzed for driver identification [5,6,7,8,12] and monitoring [9]. With the rapid development of the Internet-of-Vehicles technology and the popularization of smart terminal devices such as onboard diagnostic (OBD) devices (the OBD-II protocol provides data from the vehicle ECU ), multi-dimensional CAN-BUS data can be easily captured. Driving-behavior recognition is essentially a classification task based on in-vehicle CAN-BUS data. The selection of key features and combinations thereof significantly affect the accuracy of classification algorithms. During the past decades, several machine-learning algorithms have been proposed for performing driver identification. With regard to deploying the corresponding algorithms, one has multiple choices. One possible choice is smartphone applications, as smartphones can now be fully integrated with vehicles owing to automotive operating systems such as Android Auto [13], Automotive Grade Linux (AGL) [14], and Qnx Automotive OS [15]. However, in the case of mobile edge computing, the connected car behaves like an IoT device; moreover, it can request the edge server for services such as driver identification and several other driving-related services that are also offered in other environments. In this study, we consider the mobile edge computing for a connected car environment and focus on possible embedded solutions that can be deployed in edge server. Moreover, we explore state-of-the-art deep-learning algorithms, namely, fully convolutional networks-LSTM (FCN-LSTM) [8] and DeepConvRNN-Attention [7], for driver identification and its feasibility to be implemented in embedded hardware. We evaluated each algorithm with regard to its advantages and disadvantages. By addressing the issues and combining the merits of each algorithm, we propose a lightweight deep-learning solution. We further assessed both the algorithms using network pruning, which reduces the model size, thereby improving the performance of the driver-identification models. However, network pruning can reduce the model size only to a certain extent. Therefore, our proposed model outperforms even the compressed versions of existing algorithms. Additionally, with regard to absorb a greater number of classes, we effectively applied sparse learning on the fully connected layer of the proposed model, thereby achieving adaptability and further enhancing the identification performance. Finally, we successfully deployed the optimized proposed deep-learning model on NVIDIA docker to run them in a container environment using Jetson embedded platform (Xavier, Tx2, Nano). This enabled the model to be run as an instance (container), and adjust more classes incrementally using sparse learning, by multiple containers.

This paper deals with driver identification using the driver’s behavior data (in-vehicle sensors). Considering the distinctive features of driver behavior, our objective is to identify the driver of a given unseen driving data, using a lightweight deep learning model. To this end, we propose a complete end-to-end framework for driver identification, containing a feature of tackling a more significant number of classes by sparse learning. The following are the key contributions of this study:

- A lightweight deep-learning network is proposed for performing driver-behavior identification using in-vehicle CAN-BUS sensor data. Our proposed architecture outperforms state-of-the-art methods. Particularly, it exhibits higher accuracy, efficient memory usage, less computational complexity (number of floating-point operations (FLOPs) and the number of parameters), which improves the inference time.
- Our proposed architecture requires a shorter window size, such as 40 s, for driver identification, compared with previous research, which required at least 60 s time-series data to perform the classification.
- We study the impact of window size(number of time steps) and degree of overlap by sliding window on accuracy and computational complexity, determining the optimal values for our network.
- To further validate the effectiveness of our lightweight model, we also evaluated the current research methods by applying channel pruning at different layers to make them lightweight. We assess the optimal extent of pruning without significantly compromising the accuracy of existing models. Nevertheless, the proposed solution obtains a more compact size compared to that of existing methods by introducing depthwise convolutions. We presented a detail results in terms of inference time and memory usage at Jetson embedded system (Xavier, TX2, and Nano).
- For robust testing of our proposed model, we introduce anomalous data at different time sequences and finally apply anomaly detection method (one-class support vector machine). We presented a comparison of robustness with existing algorithms.
- To sustain the lightweight model and adjust new classes without affecting the accuracy, we make our proposed solution adaptable for new classes by developing a state-of-the-art sparse-learning technique at the fully connected layer. Accordingly, we carefully select the relatively substantial nodes for existing classes, freeze them, and re-train our network by improving the weaker nodes to classify new classes. This ensures that a high accuracy is sustained for existing classes, thereby providing room to adjust new classes without affecting the network size.
- We deployed the proposed algorithm equipped with sparse learning in the container environment of NVIDIA-Docker using the Jetson platform (Xavier, Tx2, and Nano). In this regard, our proposed model acts like active instance (container) and supports incremental learning to absorb a greater number of classes. This makes our model favorable candidate to deploy in real-time conditions (container is a virtualization method gradually becoming a base environment for edge computing applications).

## 2. Related Work

Driver-behavior profiling using driving information is an emerging trend in multiple markets, including user-based insurance and traffic safety and monitoring. It is a prolific research area with an ample number of studies. In this section, we describe the existing studies based on three primary categories: data source, machine-learning models, and applied platform.

### 2.1. Data Source

Driving information can be captured using several different sources, which mainly include in-vehicle sensors and smartphone sensors. In References [5,6,7,8,10,12], the authors utilized CAN-Bus data for driver identification. The car sensors communicated via CAN-BUS (OBD-II protocol), and the CAN-Bus data could be acquired using the OBD-II adapter. The in-vehicle sensor data included parameters related to the engine (e.g., engine coolant temperature and friction torque), fuel (e.g., long-term fuel trim bank and fuel consumption), and transmission (e.g., wheel velocity and transmission oil temperature). Similarly, in other studies [11,16,17], the authors used smartphone sensor data for driver-behavior profiling and many other applications [9]. Smartphones are equipped with sensors including GPS sensors, accelerometers, magnetometers, and gyroscopes, all of which can provide information regarding speed, acceleration, rotational speed, and several other combinations of parameters used for driver profiling. However, some researchers are emphasizing a hybrid approach based on combining vision (camera) and other sensors (LiDAR, GPS, IMU, etc.) for learning driver behavior [18,19,20] under complex situations such as those entailing goal-oriented action, stimulus-driven action, and cause-attention (e.g., stop a vehicle to let a pedestrian pass (cause) and analyze how the driver attends the situation (attention)). For the driver monitoring applications, several researchers have utilized camera video/image only [21,22]. Some other works employed physiological sensor data from bio-signals to identify a distracted driver [23]. Among the data obtained from different data sources, CAN-BUS data are the most reliable, feasible, and widely used for driver-behavior profiling [10]. The security dataset [5] provides up to 51 features captured using the CAN-BUS data; furthermore, it has been used by several researchers for driver identification [7,8]. In this study, we have used the same security dataset for driver identification.

### 2.2. Machine-Learning Models

Studies that focused on the modeling of individual driver behaviors used many state-of-the-art machine-learning models. These include statistical classification algorithms such as decision trees, random forests, k-nearest neighbors [5], hidden Markov model [24], Gaussian mixture models [25], k-means clustering [10,26], and support vector machines [26]. However, most of them suffered from various shortcomings, such as data dependency and the limitation of working under specific conditions only, which were overcome by the robust nature of deep-learning algorithms [7], which offer a significant advantage with regard to feature learning. Driver recognition using sensor data can be considered as time-series classification problem as scalar data from vehicle sensor contains temporal information in sequential manner. Therefore, deep learning frameworks for time-series classification can be explored, which are as follows.

#### 2.2.1. CNN-RNN Architectures

**Figure 1:**
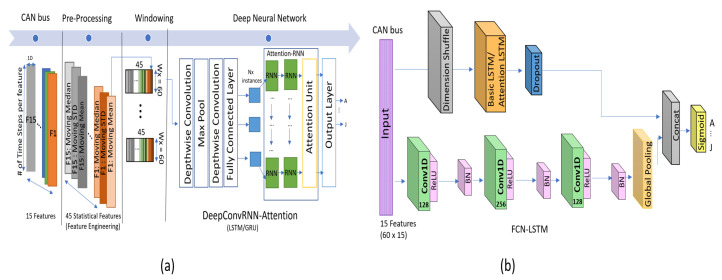
State-of-the-art deep-learning architectures for driver identification trained using the OCS Lab security dataset(CAN bus), (**a**) DeepConvRNN-Attention [7], and (**b**) FCN-LSTM [8].

Standard convolutional neural network (CNN) layers have shown promising results for extracting dense spatial features from data such as 2D images [27]. Recurrent neural networks (RNNs) are widely used for sequential-data processing, such as natural-language processing, and other time-series processing problems, to learn temporal dependencies [28]. Over the past few years, CNNs have been employed as powerful techniques to apply at a temporal dimension of the sensor data, along with pooling operations for human-activity recognition [29] by using multivariate time-series data. Similarly, multi-scale CNNs [30], and fully convolutional networks (FCN) [31] are used for end-to-end, univariate time-series classification. However, for more promising temporal feature extraction, the combination of CNNs and RNNs has offered remarkable results, such as wearable activity recognition [29], defect recognition [32], and driver-behavior identification [7], in the state-of-the-art research. All these combinations mostly contain CNNs followed by RNNs in sequential order (refer to Figure 1a). On the other hand, the augmentation of the LSTM layer is not sequential in the case of FCN-LSTM (refer to Figure 1b); moreover, it has improved the performance of FCNs with regard to time-series classification [31]. However, these combinations can vary depending on the temporal modeling problem and the nature of the dataset. In this study, we explore these two combinations, employed in state-of-the-art algorithms, for a similar task, which is “driver-behavior identification.” For better visualization, we consider these structures as being in series in the case of DeepConvRNN [29] and in parallel in the case of FCN-LSTM [31]. Recently, researchers have exploited each of these structures in (i) DeepConvRNN-Attention [7] and (ii)FCN-LSTM [8] for driver-behavior identification. We study the parameter optimization of both the algorithms to make them lightweight and adaptable by evaluating their advantages and disadvantages. Finally, we propose a novel deep-learning model that is lighter than the state-of-the-art models in addition to outperforming them in terms of inference time and accuracy.

It is to clarify that, FCN-LSTM and DeepConvRNN-Attention are the independent studies, focused to provide high-end accuracy on the driving dataset. We opted to choose these studies, due to the reason that, they have used the same dataset [5] and are considered to be the best candidates to achieve higher accuracy in recent years. We further evaluate them in terms of parameter utilization. Before explaining the proposed architecture, we first provide the details of these models accordingly.

#### 2.2.2. Driver Identification Using DeepConvRNN-Attention

The deep learning architecture in Reference [7] comprises a convolutional layer followed by attention-based RNNs in sequential order, as depicted in Figure 1a. For driver-behavior identification, the authors utilized a popular architecture, DeepConvRNN, previously used for similar time-series classification applications, such as human-activity recognition [29]; furthermore, the author significantly improved the classification accuracy by adding attention mechanism at the end of RNNs, thereby justifying the name of the proposed model, which is DeepConvRNN-Attention(Attention-GRU/Attention-LSTM). The model was trained using the Ocslab [5] security driving dataset for performing driver profiling and identification. The authors utilized 15 substantial features that were further processed with statistical (mean, median, and standard deviation) features. For statistical feature processing, they used a window size of (60 × 1) with a stride of 1; using which, they created a 45-dimensional feature (15 × 3) set whose size was the same as that of the original features set, as depicted in Figure 1a as a pre-processing step. The attention mechanism [33] is exploited to prioritize specific valuable feature instances for deriving class scores. In order to obtain competitive accuracy, authors have utilized two layers of RNNs with size of 128 each. Accordingly, these RNNs layers and fully connected layer contribute major portion of parameters due to sequentially adding them after depthwise convolution which itself contains less parameters. Depending upon the number of depth-multipliers of depthwise convolution, the subsequent layers can drastically increase the parameters if added in sequential order as shown in Figure 1a.

#### 2.2.3. Driver Identification Using FCN-LSTM

In Reference [8], the authors utilized FCN-LSTM [31] for driver-behavior profiling. Previously, the FCN-LSTM [31] was proposed for time-series-sequence classification, and it achieved remarkable results with minimum data pre-processing. The authors in [8], successfully avoided all the feature engineering (moving mean, std, and median) using FCN-LSTM, utilizing only lightweight pre-processing (normalization only) for driver-behavior classification. The deep-learning framework comprises a fully convolutional block augmented by an LSTM block, followed by a dropout, as depicted in Figure 1b. The fully convolutional block further contains three stacked temporal convolutional [34] layers with the kernel size of 128, 256, and 128, respectively. For driver classification, the input is a set of 60 × 15-sized batches, where 60 represents the time-step length and 15 the number of driving features. The CNN layers receive data as univariate time series with multiple steps (60 × 15), whereas the LSTM block receives the input as multivariate time series(15 × 60) that has N (15) variables with a single time step. This is achieved by applying dimensional shuffling before the LSTM block, thereby achieving convergence in less number of iterations. Accordingly, the competitive accuracy can be achieved using fewer number of hidden neurons in LSTM layer that is, 10 neurons in this case, which further reduces the size of the network significantly. The FCN-LSTM [8] utilized 3 times less parameters than that of DeepConvRNN-Attention [7] with slightly less accuracy, which inspired us to use FCN-LSTM and further improve the accuracy and reduce more parameters accordingly.

### 2.3. Applied Platform

With regard to deploying driver-behavior-profiling algorithms for real-time applications, there exist multiple options, such as the use of smartphones integrated with vehicles (e.g., AGL [8,14]), in-vehicle dedicated embedded computers (e.g., advanced driver-assisted systems [35]), and cloud- or edge-based services in a connected car ecosystem [36]. This study broadly consider the application of driver identification algorithms on an edge server under the umbrella of mobile edge computing. Therefore, the scope of this study is to demonstrate the implementation of the proposed method in a container environment (i.e., using NVIDIA docker), which is one of the virtualization methods gradually becoming a base environment for edge computing [37,38]. For the effective deployment of deep learning models, the market offers numerous dedicated low-power, energy-efficient embedded system solutions. Among the competitive embedded solutions, NVIDIA Jetson is the most popular, as it offers a wide range of developer kits (e.g., CUDA Toolkit and CuDNN) that offer various specifications. The NVIDIA Jetson series has many distinct features, including energy efficiency, low weight, compact form-factor, high performance per watt, and low-power GPU cores [39]. According to the analysis presented in Reference [40], Jetson provides a higher peak performance than those of Raspberry Pi and Intel Movidius (Neural Compute Stick). We chose the Jetson series for deploying the proposed driver-behavior-identification framework on Jetson platform (Xavier, Tx2, and Nano).

## 3. Methodology

In this section, we provide an overview of the different methods and platforms that have been used in this study. We start with a brief description of the problem statement, and the datasets used in the experiments. Subsequently, we present the detail of proposed deep-learning algorithm used for classification. Finally, we explain the proposed optimization methods and deployment environment used in the experiments.

### 3.1. Problem Formulation

If the driver-profiling algorithm is deployed in a real-time situation entailing a connected car environment, the driver will be identified in more than 60 s because the algorithm must wait to complete a single frame requirement (T*_w_*). The first possible, total time(T*_total_*) to infer the classification is presented by Equation (1), where T*_w_* is the time based on window size required by an algorithm, T*_p_* is the time for preprocessing (i.e, feature engineering time (moving mean, std, and median)), and T*_i_* is the inference time by classification algorithm (usually less than 1 s).

(1) $$T_{total}=T_{w}+T_{p}+T_{i}.$$

Although other algorithms [8] do not require feature engineering, they still require a window of 60 s to process time-series data for driver classification. The window size of time steps should be reduced for early identification of the driver. However, in the case of a connected car environment, where multiple cars are connected to edge servers and require fast processing, lightweight algorithms must be developed for real-time applications to fulfill requests in a short time with high accuracy. Similarly, with the development of an edge-computing environment with container orchestration (e.g., Kubernetes and SWAM [37,38]), deep-learning frameworks must be made more adaptable to the new environment via sparse learning and other techniques for new classes without losing significant accuracy on the existing classes. In this research, we have addressed aforementioned issues by introducing light-weight deep learning model equipped with sparse learning to absorb greater number of classes in real-time situation. The detail of our proposed model will be provided in subsequent sections.

### 3.2. OCS Lab—Security Driving Dataset

It is an open-sourced dataset generated by Byung Il Kwak, et al. [5] and is freely available online [41]. The dataset provides in-vehicle CAN-BUS sensor data that contain the driving information of 10 different drivers (A–J) who have followed the same path. For data acquisition, every driver performed four trips (two round trips) using the same model car manufactured by KIA Motors Corporation. The track contained different road types (city way, motorway, and parking lot) in the city of Seoul, South Korea. The total length of the trip was approximately 23 Km. The data comprise 51 features extracted from the car ECU acquired through the OBD-II protocol with the frequency of 1 Hz. The dataset holds 94,401 records and was used in Information Protection R&D Data Challenge 2018–19 under the section “Theft detection based on vehicle driving data” [42]. Moreover, in the dataset paper [5], the authors shortlisted 15 substantial features out of 51 for driver identification and proposed statistical machine-learning models for driver classification. The feature selection method by the authors reported in Reference [5] is based on the InfoGainAttributeEval evaluation method, which is previously implemented in Weka [43]. This is one of the ranker search methods for feature selection. Several researchers have utilized these shortlisted features from the dataset for driver-behavior identification [7,8]. Therefore, our proposed model also utilized the same selected features as list in the Table 1 for driver behavior identification.

Unlike other studies [5,7], where extensive feature engineering is performed as a pre-processsing step, we perform data normalization only, prior to fed our network for classification. The formula to compute normalization step is presented in Equation (2), where max(x${}_{n}$) and min(x${}_{n}$) are the maximum and minimum values present in the corresponding column of feature, and X${}_{n}$ is the normalized variable.

### 3.3. Our Proposed Framework

**Figure 2:**
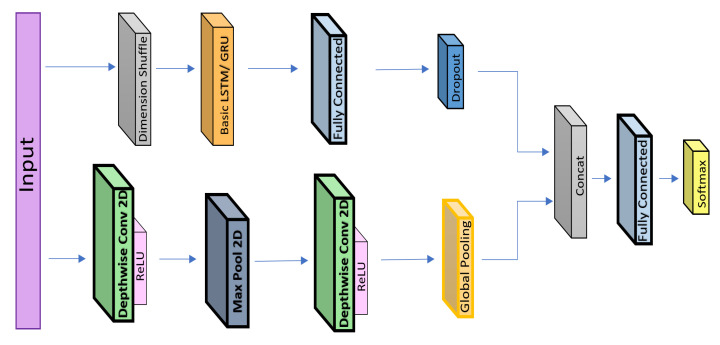
Proposed framework for driver identification.

With the motivation to build a lightweight deep-learning model, we propose to replace the 1D CNN with 2D depth-wise convolutional layers in the same configuration as that in the FCN-LSTM [8]. We successfully achieve state-of-the-art performance with significantly reduced number of parameters. The details of the proposed deep-learning architecture are shown in Figure 2. Unlike other algorithms that use 60 s of data, our proposed model employs only 40 s of driving sequence data from the Ocslab dataset [5] and outputs the classifications scores for the identification of 10 drivers (A–J). Our framework comprises a depthwise separable convolutional layer augmented by an RNN. In the case of the RNN, both the gated recurrent unit (GRU) and LSTM are evaluated for performance comparison. Each layer of the architecture is explained in the following section.

(2) $$X_{n}=\frac{x_{n}-min\left(x_{n}\right)}{max\left(x_{n}\right)-min\left(x_{n}\right)}.$$

#### 3.3.1. Depthwise Convolution

Depthwise-separable convolutions were efficiently utilized in Reference [44] to build lightweight deep neural networks. The computation cost of depthwise convolution is represented by Equation (3), where M denotes the number of input channels and D_k_ the size of the kernel that produces the output feature map of size D_F_. However, in the case of standard convolution, the computational cost is N times than that for depthwise convolution, where N denotes the number of output channels.

(3) $$D_{k}\cdot D_{k}\cdot M\cdot D_{F}\cdot D_{F}.$$

Our model applies depthwise convolutions on time series. The input size is W_T_ × F_N_, where W_T_ denotes length of time steps (40 s here) and F_N_ the number of features (15 here). The number of input channels is equal to F_N_ so that convolutions can be applied on each channel separately upon receiving data as univariate time series with multiple time steps. Each group of outputs of the convolution layer corresponds to the feature map. The optimum results were achieved using a rectified linear unit (ReLU) as the activation function for the convolution layers. The values of depth-multiplier are set to 20 and 10 to produce output channels equal to the filters_in * depth-multiplier for the first and second convolutional layers, respectively.

Similarly, the kernel sizes for the first and second depthwise convolutional layers are 9 × 1 and 5 × 1, respectively. However, to control the size of the network within a feasible range, a max-pooling layer is introduced between the depthwise convolution layers, as depicted in Figure 2. The kernel size is set to 7 × 1 with the stride of 1 × 2, thereby reducing the size and to overcome the over-fitting problem. The depthwise convolution layer was also used for similar applications in DeepConvRNN-Attention. However, the size of the network drastically increases because of adding × 2 LSTM layers (each having 128 hidden states) in sequential order (refer to Figure 1a). In our case, we replaced the 1D CNN with a depthwise layer whose configuration was similar to that of FCN-LSTM, and the LSTM layers were not augmented in sequential order (refer to Figure 1b). Subsequently, the LSTM/GRU layers of the proposed network also contain ten hidden neurons similar to FCN-LSTM.

#### 3.3.2. Recurrent Neural Networks

RNNs belong to the class of neural networks that are used to extract temporal behavior from sequential data. Standard RNNs exhibit the vanishing gradient problem when long-term sequential data are employed. This problem was solved using LSTMs by adding gating (input, output, and forget) functions and a memory unit using which the memory of the previous states could be easily controlled at each time step. Similarly, GRUs also adopt gating concepts with update gates. In our proposed model, we have utilized both LSTM and GRU layers, as depicted in Figure 2. The accuracy obtained using GRU is high for time-series data in the case of driver profiling. For a particular dataset, GRU outperformed LSTM in other studies as well [7]. In the proposed framework, the LSTM/GRU receives multivariate time-series data, which are obtained via dimension shuffling before the LSTM. An example of such data is data with 15 features (multivariate) with a single time step being associated with each set of features. In this regard, LSTM/GRU captures the temporal dependencies of the features in a fewer number of iterations than that without dimension shuffling [31]. Although LSTMs resolved the vanishing gradient problem, they still experienced difficulties because of the long-term dependencies in long sequences, and these difficulties were overcome using attention mechanisms [33]. The attention mechanism allows to focus on substantial features and helps to increase the accuracy. Accordingly, we explored the attention-based LSTM layer, which, in our case, did not perform better than the basic LSTM layer in terms of accuracy.As our model resembles the FCN-LSTM, where, they have also reported to achieve better results using basic LSTM than attention-based LSTM in similar configuration. However, in several studies related to time-series classification, attention mechanisms increased the accuracy when the augmentation of the LSTM layer was sequential, similar to that in DeepConvRNN-attention. Moreover, an attention-based LSTM layer is computationally more expensive than a basic LSTM, and contrarily, we aim to develop a lightweight solution. In summary, in our proposed architecture, the LSTM layer provides more accuracy than that of the attention-based LSTM, and GRU outperforms both of them. Additionally, the GRU requires fewer parameters than LSTM because it has fewer gate functions (only update gate). In driver-behavior identification, we successfully achieved the highest accuracy by employing GRU on a particular dataset using conditions that consumed low computational power.

#### 3.3.3. Hyperparameter Optimization

**Figure 3:**
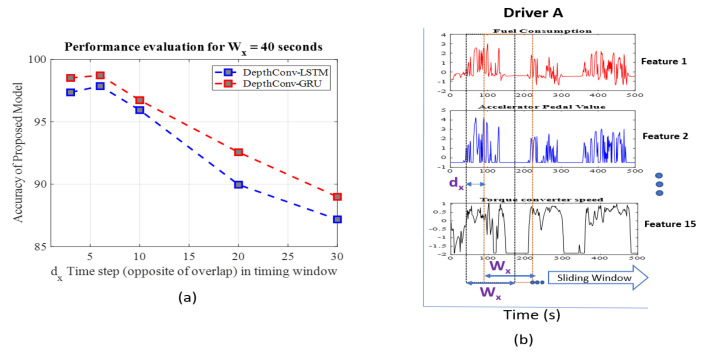
(**a**) Performance evaluation of proposed method for fixed W_x_= 40s, (**b**) Visualization of windowing operation, where W_x_ is time series size, and d_x_ is the shift between consecutive sliding window.

The data-segmentation or windowing step is critical to building an accurate driver-identification model. With regard to sequential or time-series data, determining the appropriate values of windowing hyperparameters (i.e., window size and degree of overlap) is the most crucial part, and it directly affects the model accuracy [45,46]. Hyperparameters, including the number of layers, kernel sizes in each layer, were explained in the previous Section 3.3.1. Here, we focus on the importance of window size and overlap. The input to the model depends on the window size(Wx), while the degree of overlap determines the prior information in the sequential data. The shift value dx is opposite to degree of overlap, which can be computed by using formula as overlap = Wx-dx, as depicted in Figure 3b. A study [7] was conducted to select the optimum values of both the Wx (90, 60, etc.) and dx (45, 10, 6, etc.); the best driver-identification performance was achieved for the window size (Wx) of 60 s and shift (dx) of 6 s, as shown in Table 2. Similarly, in FCN-LSTM, the authors selected the window size(Wx) as 60 and dx as 10. Our proposed model, being more compact than other algorithms, requires the values of these hyperparameters to be optimal to compete with the performances of other models. The best values were observed when the window size (Wx) was set to 40 s with a high degree of overlap (shift value of dx = 6 means overlap is 34, that is, 40 − 6 = 34), as presented in Table 2. Notably, a higher degree of overlap does not contribute to the network size; however, it affects the performance as shown in Figure 3a. We also evaluated our results for the window size(Wx) of 60 s and achieved optimum results when the value of dx is set to 10. However, the best value of dx is found to be 6, for window size (Wx) of 40 s. The visualization of windowing operation is shown in Figure 3b, where data from a particular driver A is segmented over time series using sliding window operation.

## 4. Performance Evaluation

### 4.1. Experimental Setup

For our experiments, we have used NVIDIA Jetson platform(Xavier, TX2, and Nano). Jetson Xavier is equipped with GPU (512-core Volta with Tensor Core), CPU (8-core ARMv8.2), and RAM (16 GB LPDDR4). Similarly, the TX2 and Nano are replete with GPU of 256-core(Pascal) and 128-core (Maxell) while RAM of values 8GB and 4GB, respectively. The respective embedded hardware is only used for deployment (testing dataset execution) for most of the experiments. A detailed benchmarking of these embedded hardware for numerous parameter indices is already performed in one of our previous work [47]. All experiments are executed in container environment using NVIDIA docker image. However, for training of all models, we have used desktop computer equipped with CPU (core i7-9700), GPU (GeForce RTX 2060) and RAM of 16GB, respectively. In case of sparse learning experiments (Section 5), we have utilized the Jetson platform using container environment. For this we have used multiple instances of container for incremental learning for new classes as detail is provided in Section 5, while visualization of deployment is shown in Figure 5. We have used TensorFlow(Keras) to develop all codes. Furthermore, in our experiments, we have also used sklearn, pandas, matplot-lib, numpy and other useful python libraries for data processing.

### 4.2. Cross Validation of Time Series Data

We have utilized 5-Fold cross validation, in which the data was divided into five parts, where four of them were used for training and the remaining one was employed for validation. The accuracy used in the experiments is defined by Equation (4), where TP is number of true positives, TN is the number of true negatives, FP is the number of false positives and FN is the number of false negatives, respectively.

(4) $$Accuracy=\frac{TP+TN}{TP+TN+FP+FN}.$$

The accuracy refers to the classification accuracy of the drivers, how the model predicts (driver identification) drivers based on time-series CAN-BUS sensors data.

### 4.3. Computational Complexity of the Proposed Model

The proposed deep-learning framework performs efficiently by using only a few parameters. It outperforms the state-of-the-art algorithms, as confirmed in Table 2. It achieves an accuracy of 98.72% with only 0.232 million FLOPs while employing only 119,352 parameters when using GRU as an RNN unit. Similarly, using LSTM as an RNN unit, the proposed algorithm obtained an accuracy of 97.86% by using only 119,832 parameters with 0.233 million FLOPs. Furthermore, our proposed algorithm outperforms other state-of the-art algorithms in terms of accuracy by consuming two times fewer FLOPs than those consumed by FCN-LSTM; furthermore, fewer parameters such as FCN-LSTM consumes 284,294 parameters with basic LSTM (their codes are available on GitHub [48]). DeepConvGRU-Attention achieved an accuracy of 98.36% by consuming 1.631 million FLOPs, which is seven times more than those used by the proposed framework. Because our proposed framework resembles FCN-LSTM with modifications, such as, the 1D CNNs are replaced by Depthwise convolution (Section 3.3.1), here, we briefly compare our parameters. The 1D CNN in FCN-LSTM with 128, 256, and 128 filters entails 15,488, 164,096, and 98,432 parameters, respectively. However, our proposed framework utilized only two depth-wise convolutions with depth-multipliers of 20 and 10, which consume 3000 and 18,000 parameters, respectively. Although it sounds trivial to just replace the convolution layers, nevertheless, for fine-tuning the network to achieve a competitive accuracy, ample number of studies and efforts are involved in determining the optimum values for the kernel sizes and all inter-dependent parameters in the framework. In terms of memory efficiency, the model size of our proposed deep-learning framework is only 1.69 MB(MegaBytes), which is the lowest among all the other candidates, as listed in Table 2. Unlike the different algorithms mentioned in the table, which require feature engineering (moving mean, standard deviation, median, etc.), our algorithm directly consumes raw features (only normalization), similar to the case of FCN-LSTM. The proposed framework requires only a 40 s window (time steps) for inferring the driver identity, compared with others, which require at least 60 s. For the fair comparison, we also evaluated our results using 60 s window, where our proposed algorithm outperforms in terms of memory usage (1.74 MB) and computational complexity (0.235 M parameters), achieving competitive accuracy. Subsequently, with motivation to infer earlier than existing solutions, we targeted 40 s, and achieved outstanding results in all performance metrics as shown in Table 2. Like all algorithms presented in Table 2, we do not use any refinement technique, such as fine-tuning (reducing learning rate), and sparse learning at this stage to investigate the actual performance of the proposed deep-learning architecture.

The following are the key factors involved in reducing both the number of parameters and size of the proposed network:

- The input size (40 × 15) is the key to reducing the network size, which mainly contributes to memory consumption. Although it does not consume parameters, it is indirectly involved in reducing the number of parameters of the subsequent layers.
- A max-pooling layer is utilized to effectively reduce the network size (memory and parameters) by setting a high filter size, thereby alleviating the over-fitting problem. This, in turn, indirectly affects the parameters of subsequent layers.
- Depthwise convolution layers with large-sized filters and few depth-multipliers can effectively reduce the number of parameters.
- The augmentation of the LSTM layer is not sequential; instead, the input is separately fed to the convolutional and LSTM layers in the proposed model, thereby reducing the size of the hidden LSTM layer, such as a single layer with 10 hidden states in our case. However, DeepConvLSTM uses two LSTM layers with 128 hidden neurons each to acquire a competitive accuracy.

The proposed model outperforms the other state-of-the-art algorithms in terms of accuracy, with significantly low computational complexity (i.e., low number of FLOPs and parameters), and its most compact size is presented in Table 2. Subsequently, the proposed model outperforms in terms of inference time while running in embedded hardware (Jetson platform), as shown in Table 3.

### 4.4. Robustness to Data Anomalies

The accuracy of driver-identification algorithms is directly associated with the reliability of car sensors, which are prone to malfunction, noise, failures, and hacking attempts. Feeding the network with this kind of anomalous and erroneous data can deteriorate the model accuracy. However, even when fed with anomalous data, our network outperforms other networks, thereby demonstrating its robustness (Table 4). Our robustness test is inspired by FCN-LSTM [8]. In this regard, n random sensors are selected to be modified out of total 15 sensors (i.e., features), where n is 7 in this case. Different rate of anomalies are simulated such that 1%, 10%, and 50% of total samples from validation set were modified by random values in 7 features each (Table 4). Similarly, for each rate, two anomalies duration (i.e., 1 s and 10 s) were simulated. In this case, 1 s and 10 s means, we modified out of 40 s (Wx) for each input sample (40 × 15). Summarizing, 1% of anomaly means, 1% of total validation samples, while 1 s means 1 of 40 s per sample data is modified with random noise. It can be seen in Table 4, the minimum accuracy of proposed algorithm (Depthconv-LSTM), shortly DC-LSTM, obtained as 75.21% even when introduced with 50% anomalies. Similarly, proposed DC-GRU obtained minimum of 75.43% accuracy even in the presence of 50% of anomalies. Our proposed solution outperforms the FCN-LSTM. Moreover, we have used outlier rejection algorithm, the one-class support vector machine(SVM), to correct the anomalies. Previously, one-class SVM was found to be the best candidate for anomaly detection, after detailed comparison [8]. One-Class svm identifies anomalies, which can be used for correction and estimation of original data. In this regard, the trivial method is to take average of neighbors. For instance, given the order sequence of any feature values {10,3000,20}, recorded at timestamps of t, t+1, t+2 after anomalies injection. Accordingly, the one-class SVM will identify, t+1 location, as outlier, and to remove anomaly, we replace the values of t+1 with average (i.e., 15). The detailed results are shown in Table 4. Our proposed model is more robust to anomalies than FCN-LSTM, which make it a favorable candidate to deploy in real-time conditions.

### 4.5. Comparison with Compressed versions of Existing Models

**Figure 4:**
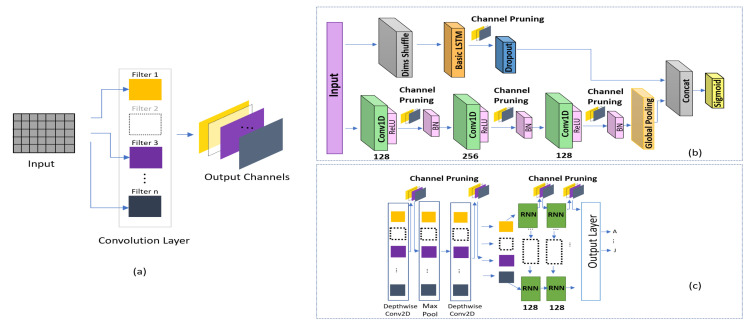
(**a**) Channel pruning. Locations where channel pruning was applied in (**b**) FCN-LSTM and (**c**) DeepConvLSTM-Attention.

This study primarily aims to produce a lightweight solution. Most studies in the literature focus on compression methods including network pruning, quantization, Huffman coding [49], and sparse-regularization [50] of the existing architectures to remove unnecessary connections and make the neural network more compact without compromising the accuracy. We have used depthwise convolution to reduce the architecture size; however, the existing models can be compressed to produce a lightweight solution. In addition, compression is easier than significantly changing the architecture. Accordingly, we compare our results with those obtained using the compressed versions of state-of-the-art frameworks in terms of driver identification. We successfully applied channel pruning on each layer of DeepConvRNN-Attention and FCN-LSTM as shown in Figure 4b. In Reference [51], it was reported that channel pruning was more sensitive in the initial and last layers. However, the layers in the middle were less affected in terms of accuracy, indicating a possibility for further pruning while maintaining the accuracy. To select unimportant channels to prune, we followed a well-known L1-norm-based method [51]. The following are the steps for channel pruning.

- For each filter in a particular layer, compute the sum of its absolute kernel weights using equation of L1 norm [51].
- Sort the filter on the basis of the L1 norm values calculated in the previous step.
- Prune the m filters that have the smallest L1 norm values, and remove the corresponding feature maps from the current and next layers.
  – To select the optimum values of the m filters to be pruned, we begin pruning with the smallest number, that is, 10, and start evaluating the accuracy on a validation dataset.
- To delete the channels from layer i and i+1, we use the Keras-surgeon [52] tool, which deletes the channels and provides a new copy of the pruned model.

The channel pruning performed is visualized in Figure 4a. We applied channel pruning on all the three 1D constitutional layers and LSTM layer of FCN-LSTM, as depicted in Figure 4b. Similarly, we pruned the filters of both the depthwise convolution layers and two LSTM layers of DeepConvLSTM-Attention, as depicted in Figure 4c. As depthwise convolutions utilize fewer parameters, so pruning significantly affect the accuracy when applied at depthwise of DeepConvGRU-Attention. The final configuration of pruning for selected layers, the effect of the pruning on accuracy and computational complexity is summarized in Table 3. The proposed method outperforms all listed algorithms in terms of accuracy, memory usage, and inference time in all Jetson platform.

## 5. Deploying the Proposed Model with Sparse Learning

### 5.1. Sparse Learning

Sparse learning is a technique in which a pre-trained network is analyzed to categorize the weaker and the strong nodes among layers, which may exist in scattered locations. The best nodes from all or selected layers are then frozen (non-trainable), and the weaker nodes in selected layers are re-trained. During training, the weaker nodes are sparsely located among different layers, which is the reason to regard it as sparse learning. In case of transfer learning, network adopts new classes or tasks by using the knowledge of an existing weights without training from scratch [53]. However, in sparse learning, only the connections of the selected nodes are trained when a new class is considered [54]. The training method in sparse learning comprises two steps: freezing the non-target parameters (important nodes for the existing classes) and performing conventional training on the selected parameters (weak nodes) like in the case of transfer learning for new classes.

### 5.2. Node Selection Towards Sparse Learning

**Figure 5:**
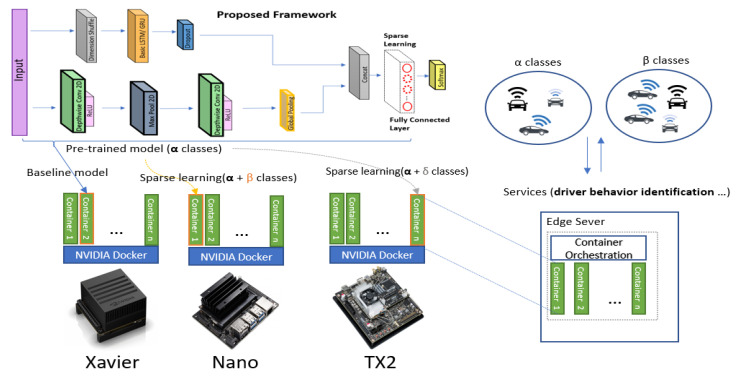
Visualization of sparse-learning deployment. Our implementation focuses on the container environment only for data.

**Figure 6:**
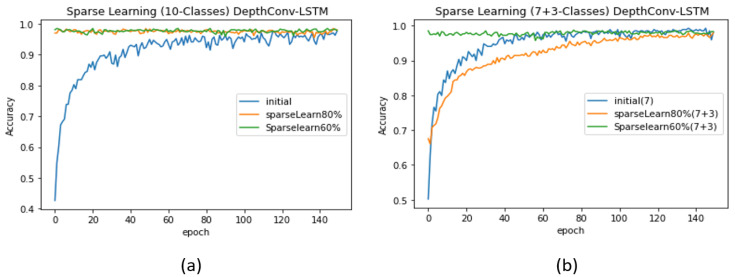
Performance evaluation (DepthConv-LSTM) with and without sparse learning: (**a**) without introducing new classes and (**b**) with the introduction of a smaller number of classes than existing.

To train the proposed model to learn new data while retaining the performance of the existing classes without affecting the network size, we implement sparse learning on the proposed model. This enables our lightweight model to adapt to a greater number of classes without compromising the accuracy and resources (parameters) with regard to the existing categories. Accordingly, we first shortlist the strong nodes in the fully connected layers using two node-selection methods: (i) ranking by the magnitude of nodes and (ii) the average activation method [54]. Thus, we deploy the proposed adaptable model in a container environment, as depicted in Figure 5. To validate our idea, we first perform sparse learning without introducing new class data, as depicted in Table 5, that is, we employ 10 classes in the case of the initial network, with the test accuracy of 97.86%. We then freeze 20% of the nodes (20% of 32 nodes is 7 nodes) via ranking by the magnitude of nodes. We retrain our network to further finetune the weaker nodes (the remaining 28 nodes), thereby increasing the accuracy by up to 0.86%. Similarly, the accuracy increased upon freezing 40% of the nodes (i.e., 40% of 32 nodes is 13 nodes). The test accuracy is listed in Table 5. The detailed pattern of the validation accuracy corresponding to each case is depicted in Figure 6a. The increase in the performance via sparse learning, when no new data are introduced to the model, shows that more classes can be adjusted. Because the number of categories is limited (maximum 10) in a particular dataset (Ocslab), we could only generate cases within 10 classes. Thus, we attempted to mimic the real environment wherein hundreds of classes would be available for sparse learning.

The sparse learning conducted to adjust the new classes can be further divided into two cases. Here, we assume that α is the number of existing classes and β the number of new classes.

**Figure 7:**
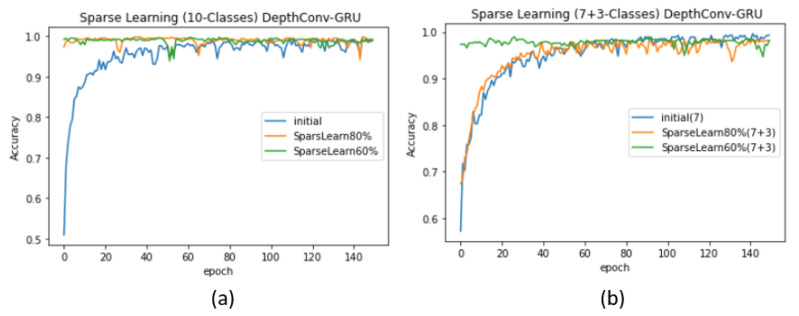
Performance evaluation (DepthConv-GRU) with and without sparse learning: (**a**) without introducing new classes and (**b**) with the introduction of a smaller number of classes than existing.

**Figure 8:**
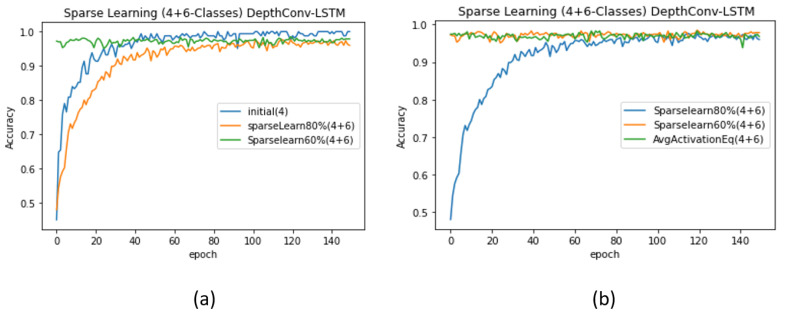
Performance evaluation (DepthConv-LSTM) upon introducing more classes than the existing number of classes (**a**) using the ranking of nodes, (**b**) comparison of the ranking of nodes obtained using the average activation method.

**Figure 9:**
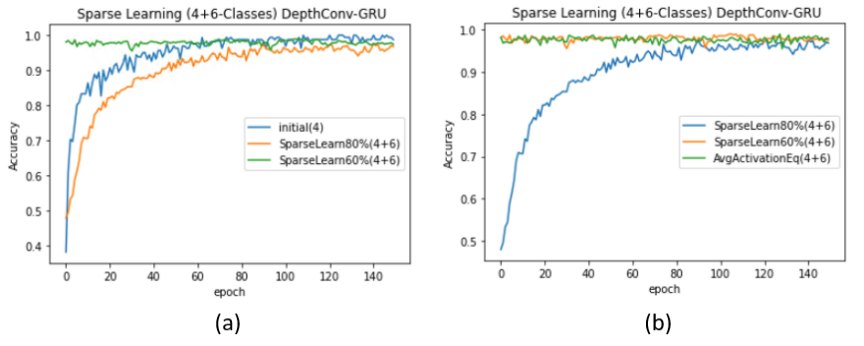
Performance evaluation (DepthConv-GRU) upon introducing more classes than the existing number of classes (**a**) using the ranking of nodes, (**b**) comparison of the ranking of nodes obtained using the average activation method.

- Case 1 (α > β): As a proof of concept, we begin with seven (α = 7) randomly selected classes (for simplicity, we consider A–G) and train the model from scratch (see Table 5) to obtain an initial network with a significantly high test accuracy of 99.11%. The complete pattern exhibited by the validation accuracy is depicted in Figure 7b. First, we freeze 20% of the nodes in the pre-trained network for α classes. Later, we feed the network with the data of α+β (7 + 3) classes and retrain the 80% of weak nodes for fine tuning. The test accuracy decreases to 97.86%, similar to that in the case of training 10 classes from scratch. However, upon freezing 40% of the nodes and re-training only 60% of the nodes, the test accuracy becomes significantly high (see Table 5). Such sparse learning can result in higher accuracy than that in the original training procedure of starting from the scratch. Another supporting argument is the pattern followed by the validation accuracy: when we retrain 80% of the nodes, the validation accuracy of the model starts from the seventies, whereas when we retrain only 60% nodes, the validation accuracy for the first few epoch starts from the nineties (see Figure 7b). Similar pattern can be found in case of DepthConv-GRU as shown in Figure 9a,b.
- Case 2 (α < β): We repeat similar experiments for the case where there are more new classes (β) than existing classes (α). However, in practice, this case can degrade the network performance [54]. This is because the pre-trained model can find difficulties in adjusting to an enormous amount of data as compared with the small amount of data previously used for training; thus, it may require major weight updates to achieve an acceptable accuracy. To resolve this issue, the authors in [54] proposed the following formula as an effective node-selection method:
  (5) $$0<A_{avg}^{\left(i\right)}\leq \mu -\left(1-log\left(1+\frac{\beta }{\alpha }\right)\right)\sqrt{\frac{1}{M}\Sigma_{0}^{M}{\left(A_{avg}^{\left(i\right)}-\mu \right)}^{2}}.$$
  This formula was previously validated on LeNet, AlexNet, and VGGNet to adapt to up to 40% new classes without a significant loss in the accuracy corresponding to existing classes [54]. We exploited this formula for node selection in the fully connected layer of the proposed model. We retrained the nodes whose magnitudes ranged from 0 to the value obtained using the formula. Because all the nodes values were activated using ReLU, their magnitudes were greater or equal to zero. Accordingly, we retrained all the nodes that lied in this range and froze the remaining ones. When α = 4, we obtained the initial test accuracy of 99.51% upon training only 4 classes (see Table 5). However, upon freezing the nodes according to the formula and feeding the network with data regarding α+β(4 + 6) classes, the accuracy suddenly dropped to 96.79%, which is lower than the original accuracy (97.86%) of the network when trained from scratch. Similarly, upon freezing 20% nodes using the previous ranking-by-magnitude method, we obtained a lower accuracy, that is, 96.37%. We achieved an accuracy of 97.86% upon freezing 40% nodes and re-training the rest using sparse learning. The corresponding validation accuracy curves for DepthConv-LSTM are depicted in Figure 8a,b. Similar pattern can be found in case of DepthConv-GRU as shown in Figure 9a,b.

After training the network, we froze the best nodes of the last fully connected layer, as depicted in Figure 5 (see the dotted line). Each container could download the baseline model (the proposed adaptable model) and add new classes using sparse learning. Our container-based solution can be later deployed in the edge sever, where any type of container-orchestration method, such as Kubernetes or SWAM, can use the container as the fundamental block [37,38].The location of the edge server will be closed to the client (i.e., car in our case), and can be accessed through authorization (i.e., token, client certification) based on container orchestration system (i.e., Kubernetes, SWAM, etc.).

## 6. Discussion and Conclusions

The proposed model is a lightweight deep-learning solution for driver identification. Notably, it is more compact than the state-of-the-art algorithms and outperforms them in terms of accuracy (see Table 2).

Compared with the state-of-the-art algorithms, the proposed solution is more robust to anomalies, thereby providing a better solution in real-time situations in the case of any malicious noise in sensors or the communication of CAN bus data. Moreover, our algorithm is equipped with a sparse-learning feature that enables it to absorb a more significant number of classes than that by traditional techniques in the container environment for driver-behavior identification. Furthermore, our proposed deep-learning architecture is more compact than even the compressed versions of conventional algorithms. To achieve a competitive accuracy, we fine-tune the degree of overlap in the window (i.e., the length of time steps). The overlap does not contribute to the model size but is crucial in the performance of the subsequent deep-learning architecture in time-series classification, such as driver identification in our case. The method of augmenting the RNN layer(LSTM/GRU) with a convolutional layer is also crucial in terms of the computational complexity and convergence of the deep-learning architecture for driver-behavior identification. In case of sparse learning, when existing number of classes are significantly more than new classes, we prefer ranking of magnitude technique to use as node selection method at fully connected layer. Contrarily, in case, the number of new classes are greater in number than existing classes data, we prefer to utilize average activation technique [54] as node selection method for sparse learning. We successfully deployed the proposed deep learning architecture on NVIDIA Docker to run it in a container environment using Jetson embedded platform (Xavier, Tx2, Nano). Subsequently, the proposed model can be run as an instance(container), and adjust a greater number of classes incrementally using sparse learning, by multiple containers in a real-time container environment at edge server, under the umbrella of edge computing.

## Figures and Tables

**Table 1:** Features selected for driver identification.

Dataset	# of Selected Features	Features
Security Driving Dataset [5]	15	Long_Term_Fuel_Trim_Bank1, Intake_air_pressure, Accelerator_Pedal_value, Fuel_consumption, Torque_of_friction, Maximum_indicated_engine_torque, Engine_torque, Calculated_LOAD_value, Activation_of_Air_compressor, Engine_coolant_temperature, Transmission_oil_temperature, Wheel_velocity_front_left-hand, Wheel_velocity_front_right-hand, Wheel_velocity_rear_left-hand, Torque_converter_speed

**Table 2:** Performance Comparison of proposed framework for driver identification.

Input	Algorithm	Accuracy	FLOPs	Memory	Feature Engineering	Windowing
60 × 45	DeepConvLSTM	97.72	1.624 M	7.88 MB	Yes	Wx = 60, dx = 6
60 × 45	DeepConvGRU	95.19	1.623 M	7.88 MB	Yes	Wx = 60, dx = 6
60 × 45	DeepConvLSTM-Attention	97.86	1.632 M	7.91 MB	Yes	Wx = 60, dx = 6
60 × 45	DeepConvGRU-Attention	98.36	1.631 M	7.91 MB	Yes	Wx = 60, dx = 6
60 × 15	FCM-LSTM	95.1	0.56 M	3.28 MB	No	Wx = 60, dx = 10
60 × 15	Proposed DepthConv-LSTM	97.78	0.235 M	1.74 MB	No	Wx = 60, dx = 10
60 × 15	Proposed DepthConv- GRU	98.52	0.234 M	1.74 MB	No	Wx = 60, dx = 10
40 × 15	**Proposed DepthConv-LSTM**	**97.86**	0.233 M	1.69 MB	No	Wx = 40, dx = 6
40 × 15	**Proposed DepthConv- GRU**	**98.72**	0.232 M	1.69 MB	No	Wx = 40, dx = 6

**Table 3:** Performance comparison of Proposed Model with compressed version of competitive models.

Algorithm	Pruning	FLOPs (M)	Memory (MB)	Accuracy (%)	# of Channels Pruned/Total	Inference Time (μs/Sample) (Container-NVIDIA Docker)		
						Xavier	TX2	Nano
DeepConv GRU - Attention	0%	1.627	7.91	98.36	-	∼505	∼1175	∼2580
	8.50%	1.482	7.48	98.04	LSTM1(07/128) LSTM2(11/128)	∼469	∼1040	∼2270
	19%	1.314	6.64	96.89	LSTM1 (20/128) LSTM2(15/128)	∼452	∼997	∼2160
FCN-LSTM	0%	0.566	3.28	95.10	-	∼284	∼365	∼450
	5.30%	0.535	3.14	94.32	Conv1(10/128) Conv2(10/256)	∼253	∼342	∼416
	12.20%	0.496	3.07	93.92	Conv1(10/128) Conv2(20/256) Conv3(10/256)	∼241	∼333	∼371
Proposed DC-LSTM	0%	0.233	1.69	97.86	-	∼188	∼207	∼230
Proposed DC-GRU	0%	0.232	1.69	98.72	-	∼182	∼205	∼227

**Table 4:** Performance evaluation in the presence of Anomalous Data.

Anomaly Rate	Anomaly Duration	Accuracy with Anomalies			Corrected Anomalies (One-Class SVM)		
		Proposed DC-GRU	Proposed DC-LSTM	FCN-LSTM	Proposed DC-GRU	Proposed DC-LSTM	FCN-LSTM
0%	1 s	98.72	97.86	95.1	98.72%	97.86%	95.10%
	10 s	98.72	97.86	95.1			
1%	1 s	98.08	97.22	93.25	97.32	96.62	93.89
	10 s	98.08	97.22	92.6			
10%	1 s	92.74	92.52	85.85	97.12	96.22	93.57
	10 s	93.16	92.09	84.89			
30%	1 s	82.69	81.62	70.42	96.32	95.52	92.93
	10 s	81.2	81.62	69.77			
50%	1 s	72.86	73.5	57.23	95.14	94.51	91.64
	10 s	75.21	75.43	57.88			

**Table 5:** Performances of the proposed model before and after using sparse learning.

# of Classes	Sparse Learing only FC Layer	Proposed DepthConv-LSTM Accuracy (%)	Proposed DepthConv-GRU Accuracy (%)	Node Selection Method
10	Initial Network	97.86	98.72	-
10	80% re-train, 20% Freeze	98.72	98.72	Ranking Mg. of nodes
10	60% re-train, 40% Freeze	98.08	98.5	Ranking Mg. of nodes
7	Initial Network	99.11	99.4	-
7+3	80% re-train, 20% Freeze	97.86	99.15	Ranking Mg. of nodes
7+3	60% re-train, 40% Freeze	98.29	99.15	Ranking Mg. of nodes
4	Initial Network	99.51	99.51	-
4+6	80% re-train, 20% Freeze	96.37	98.08	Ranking Mg. of nodes
4+6	60% re-train, 40% Freeze	97.86	97.44	Ranking Mg. of nodes
4+6	re_train 0=<Av<Eq_value	96.79	98.08	Avg Activation Method

## References

[1] Carfora M.F., Martinelli F., Mercaldo F., Nardone V., Orlando A., Santone A., Vaglini G. (2019). A “pay-how-you-drive” car insurance approach through cluster analysis. Soft Comput..

[2] Troncoso C., Danezis G., Kosta E., Balasch J., Preneel B. (2010). Pripayd: Privacy-friendly pay-as-you-drive insurance. IEEE Trans. Dependable Secure Comput..

[3] Dai R., Lu Y., Ding C., Lu G. (2017). The effect of connected vehicle environment on global travel efficiency and its optimal penetration rate. J. Adv. Transp..

[4] Lee J., Kao H., Yang S. (2014). Service innovation and smart analytics for industry 4.0 and big data environment. Procedia CIRP.

[5] Kwak B.I., Woo J., Kim H.K. Know your master: Driver profiling-based anti-theft method. Proceedings of the 2016 IEEE 14th Annual Conference on Privacy, Security and Trust (PST).

[6] Kang Y.G., Park K.H., Kim H.K. (2019). Automobile theft detection by clustering owner driver data. arXiv.

[7] Zhang J., Wu Z., Li F., Xie C., Ren T., Chen J., Liu L. (2019). A deep learning framework for driving behavior identification on in-vehicle CAN-BUS sensor data. Sensors.

[8] el Mekki A., Bouhoute A., Berrada I. (2019). Improving driver identification for the next-generation of in-vehicle software systems. IEEE Trans. Veh. Technol..

[9] Júnior J.F., Carvalho E., Ferreira B.V., de Souza C., Suhara Y., Pentland A., Pessin G. (2017). Driver behavior profiling: An investigation with different smartphone sensors and machine learning. PLoS ONE.

[10] Fugiglando U., Massaro E., Santi P., Milardo S., Abida K., Stahlmann R., Netter F., Ratti C. (2018). Driving behavior analysis through CAN bus data in an uncontrolled environment. IEEE Trans. Intell. Transp. Syst..

[11] Castignani G., Derrmann T., Frank R., Engel T. (2015). Driver behavior profiling using smartphones: A low-cost platform for driver monitoring. IEEE Intell. Transp. Syst. Mag..

[12] Park K.H., Kim H.K. (2019). This car is mine!: Automobile theft countermeasure leveraging driver identification with generative adversarial networks. arXiv.

[13] Androidauto-Connect Your Phone to Car Display. https://www.android.com/auto/.

[14] (2020). Automotive Grade Linux. https://www.automotivelinux.org/.

[15] (2020). QNX in Automotive-QNX Software Systems. https://blackberry.qnx.com/en/software-solutions/connected-autonomous-vehicles.

[16] Kashevnik A., Lashkov I., Gurtov A. (2019). Methodology and mobile application for driver behavior analysis and accident prevention. IEEE Trans. Intell. Transp. Syst..

[17] Warren J., Lipkowitz J., Sokolov V. (2019). Clusters of driving behavior from observational smartphone data. IEEE Intell. Transp. Syst. Mag..

[18] Li M.G., Jiang B., Che Z., Shi X., Liu M., Meng Y., Ye J., Liu Y. DBUS: Human driving behavior understanding system. Proceedings of the IEEE International Conference on Computer Vision Workshops.

[19] Ramanishka V., Chen Y., Misu T., Saenko K. Toward driving scene understanding: A dataset for learning driver behavior and causal reasoning. Proceedings of the IEEE Conference on Computer Vision and Pattern Recognition.

[20] Fridman L., Brown D.E., Glazer M., Angell W., Dodd S., Jenik B., Terwilliger J., Kindelsberger J., Ding L., Seaman S. (2017). MIT autonomous vehicle technology study: Large-scale deep learning based analysis of driver behavior and interaction with automation. arXiv.

[21] Wijnands J.S., Thompson J., Nice K.A., Aschwanden G.D.P.A., Stevenson M. (2019). Real-time monitoring of driver drowsiness on mobile platforms using 3D neural networks. Neural Comput. Appl..

[22] Kim W., Jung W., Choi H.K. (2019). Lightweight driver monitoring system based on multi-task mobilenets. Sensors.

[23] Taamneh S., Tsiamyrtzis P., Dcosta M., Buddharaju P., Khatri A., Manser M., Ferris T., Wunderlich R., Pavlidis I. (2017). A multimodal dataset for various forms of distracted driving. Sci. Data.

[24] Zhang X., Zhao X., Rong J. (2014). A study of individual characteristics of driving behavior based on hidden Markov model. Sens. Transducers.

[25] Miyajima C., Nishiwaki Y., Ozawa K., Wakita T., Itou K., Takeda K., Itakura F. (2007). Driver modeling based on driving behavior and its evaluation in driver identification. Proc. IEEE.

[26] Van Ly M., Martin S., Trivedi M.M. Driver classification and driving style recognition using inertial sensors. Proceedings of the 2013 IEEE Intelligent Vehicles Symposium (IV).

[27] Krizhevsky A., Sutskever I., Hinton G.E. ImageNet classification with deep convolutional neural networks. Proceedings of the Advances in Neural Information Processing Systems, Harrahs adn Harverys.

[28] Greff K., Srivastava R.K., Koutník J., Steunebrink B.R., Schmidhuber J. (2016). LSTM: A search space odyssey. IEEE Trans. Neural Netw. Learn. Syst..

[29] Ha S., Choi S. Convolutional neural networks for human activity recognition using multiple accelerometer and gyroscope sensors. Proceedings of the 2016 IEEE International Joint Conference on Neural Networks (IJCNN).

[30] Cui Z., Chen W., Chen Y. (2016). Multi-scale convolutional neural networks for time series classification. arXiv.

[31] Karim F., Majumdar S., Darabi H., Chen S. (2017). LSTM fully convolutional networks for time series classification. IEEE Access.

[32] Liu T., Bao J., Wang J., Zhang Y. (2018). A hybrid CNN–LSTM algorithm for online defect recognition of CO_2_ welding. Sensors.

[33] Bahdanau D., Cho K., Bengio Y. (2014). Neural machine translation by jointly learning to align and translate. arXiv.

[34] Wang Z., Yan W., Oates T. Time series classification from scratch with deep neural networks: A strong baseline. Proceedings of the 2017 IEEE International Joint Conference on Neural Networks (IJCNN).

[35] Brookhuis K.A., de Waard D., Janssen W.H. (2001). Behavioural impacts of advanced driver assistance systems—An overview. Eur. J. Transp. Infrastruct. Res..

[36] Curry E., Sheth A. (2018). Next-generation smart environments: From system of systems to data ecosystems. IEEE Intell. Syst..

[37] Hui K., Le M., Tao S. Container and microservice driven design for cloud infrastructure devops. Proceedings of the 2016 IEEE International Conference on Cloud Engineering (IC2E).

[38] Bernstein D. (2014). Containers and cloud: From lxc to docker to kubernetes. IEEE Cloud Comput..

[39] Mittal S. (2019). A Survey on optimized implementation of deep learning models on the NVIDIA Jetson platform. J. Syst. Architect..

[40] Kim C.E., Oghaz M.M.D., Fajtl J., Argyriou V., Remagnino P. (2018). A comparison of embedded deep learning methods for person detection. arXiv.

[41] OCS Lab Driving Dataset. http://ocslab.hksecurity.net/Datasets/driving-dataset.

[42] Information Protection R&D Data Challenge 2019. http://datachallenge.kr/challenge18/vehicle/tutorial/.

[43] Hall M., Frank E., Holmes G., Pfahringer B., Reutemann P., Witten I.H. (2009). The WEKA data mining software: An update. ACM SIGKDD Explor. Newsl..

[44] Howard A.G., Zhu M., Chen B., Kalenichenko D., Wang W., Weyand T., Andreetto M., Adam H. (2017). Efficient convolutional neural networks for mobile vision applications. arXiv.

[45] Rastgoo M.N. (2019). Driver Stress Level Detection Based on Multimodal Measurements. Ph.D. Thesis.

[46] Dehghani A., Sarbishei O., Glatard T., Shihab E. (2019). A quantitative comparison of overlapping and non-overlapping sliding windows for human activity recognition using inertial sensors. Sensors.

[47] Ullah S., Kim D.H. Benchmarking Jetson Platform for 3D Point-Cloud and Hyper-Spectral Image Classification. Proceedings of the 2020 IEEE International Conference on Big Data and Smart Computing (BigComp).

[48] A Driver Identification Framework on AutoMotive Grade Linux. https://github.com/vcar/AGL.

[49] Han S., Mao H., Dally W.J. (2015). Deep compression: Compressing deep neural networks with pruning, trained quantization and Huffman coding. arXiv.

[50] Scardapane S., Comminiello D., Hussain A., Uncini A. (2017). Group sparse regularization for deep neural networks. Neurocomputing.

[51] Li H., Kadav A., Durdanovic I., Samet H., Graf H.P. (2016). Pruning filters for efficient convnets. arXiv.

[52] Keras-Surgeon, for Network Pruning Available on Github. https://github.com/BenWhetton/keras-surgeon.

[53] Quattoni A., Collins M., Darrell T. Transfer learning for image classification with sparse prototype representations. Proceedings of the 2008 IEEE Conference on Computer Vision and Pattern Recognition.

[54] Ibrokhimov B., Hur C., Kang S. (2020). Effective node selection technique towards sparse learning. Appl. Intell..

